# Comparing copromicroscopy to intestinal scraping to monitor red fox intestinal helminths with zoonotic and veterinary importance

**DOI:** 10.3389/fvets.2022.1085996

**Published:** 2023-01-12

**Authors:** Erica Marchiori, Federica Obber, Roberto Celva, Federica Marcer, Patrizia Danesi, Anna Maurizio, Lucia Cenni, Alessandro Massolo, Carlo Vittorio Citterio, Rudi Cassini

**Affiliations:** ^1^Department of Animal Medicine, Production and Health, University of Padova, Legnaro, PD, Italy; ^2^Istituto Zooprofilattico Sperimentale delle Venezie, Legnaro, PD, Italy; ^3^Ethology Unit, Department of Biology, University of Pisa, Pisa, Italy; ^4^Applied Ecology Research Unit, Research and Innovation Centre, Fondazione Edmund Mach, Trento, Italy; ^5^Conservation Genomics Research Unit, Research and Innovation Centre, Fondazione Edmund Mach, Trento, Italy; ^6^Department of Ecosystem and Public Health, Faculty of Veterinary Medicine, University of Calgary, Calgary, AB, Canada; ^7^UMR CNRS 6249 Chrono-Environnement, Université Bourgogne Franche-Comté, Besançon, France

**Keywords:** copromicroscopy, gastro-intestinal parasites, *Echinococcus multilocularis*, helminths, red fox

## Abstract

The red fox acts as reservoir for several helminthic infections which are of interest for both public and animal health. Huge efforts have been made for the assessment of the sensitivity of coprological tests for the detection of *Echinococcus multilocularis*, while less attention has been paid to other helminthic species. This study aimed at assessing the performance of two copromicroscopic techniques in the detection and prevalence estimation of gastrointestinal helminths in the red fox. Helminths were isolated from the small intestines of 150 red foxes from Bolzano province, Italy, with a scraping, filtration and counting technique (SFCT) and morphologically identified. Rectal contents were collected and submitted to simple flotation (FT) and, only for Taenids, a method based on the concentration of eggs and identification with multiplex PCR (CMPCR). Using SFCT as a reference standard, we estimated the sensitivity of the copromicroscopic tests. Three species of nematodes (namely, *Toxocara canis, Uncinaria stenocephala* and *Pterygodermatites* sp.) and five species of cestodes (*E*. *multilocularis, Taenia crassiceps, T. polycantha, Hydatigera taeniaeformis, Mesocestoides* sp.) were identified with SFCT, whereas eggs referable to the same taxa were detected with fecal diagnostics, except for *Pterygodermatites* sp. and *Mesocestoides* sp. The sensitivity of FT was low for all taxa, ranging from 9.8 to 36.3%, with lower values for Taeniidae. CMPCR was confirmed to perform better for the detection of Taeniidae eggs (23.5%) and the multiplex PCR on retrieved eggs was effective in the identification of the species. A meta-analysis of literature also suggested that our results are consistent with existing data, indicating that copromicroscopy tends to underestimate the prevalence of helminthic infections. The extent of such underestimation varies with taxon, being higher at high prevalence levels, in particular for cestodes. Irregular dynamics of egg shedding, and routine deep freezing of red fox feces may explain the frequency of false negatives with copromicroscopy. Low sensitivity of copromicroscopic tests should be accounted for when estimating prevalence and when defining the correct sample size for the detection of the parasites.

## 1. Introduction

Monitoring pathogens in wild species is particularly relevant where wildlife acts as the epidemiological reservoir of parasites with potential high impact on human and veterinary health (1). Copromicroscopy is widely employed in monitoring infections during surveillance of zoonotic helminthiases in different animal species (2–4). Contrary to necropsy-based approaches, which are necessarily associated with culling campaigns or passive surveillance plans, copromicroscopy allows the analysis of a larger number of hosts with limited logistic needs, with relatively low cost and time effort.

The role of red foxes (*Vulpes vulpes*) in transmitting some helminthic infections to domestic animals and humans gained an increasing interest, as this generalist carnivore became an “urban exploiter” with changes in its spatial distribution and behavioral ecology (5–7). Huge effort in research has been spent on *Echinococcus multilocularis*, whose metacestode larva is the causative agent of alveolar echinococcosis. Because of its geographical distribution, morbidity severity and case-fatality ratio, alveolar echinococcosis has been recently recognized as one of the most relevant food-borne parasitic zoonoses in Europe (8), and, in the European context, the red fox represents the main definitive host for this parasite (9). However, the red fox is carrier of other zoonotic helminths causing diseases mainly in immunosuppressed or socio-economically disadvantaged individuals, such as the ocular *larva migrans* and the neurotoxocariasis caused by *Toxocara canis* larvae (10–12). Percutaneous penetration of larvae of *Ancylostoma* spp. is also possible in humans (13). Nevertheless, these parasites are particularly relevant for pet dogs, for which high burdens of hookworms may cause anemia and peracute and acute disease, especially in puppies (14). The less common zoonotic Cyclophyllidea include the genera *Dipylidium* and *Mesocestoides*, which may induce patent intestinal infections in humans, with the former being more frequently reported, especially in children (15). Infection by *Mesocestoides* spp. is rather well known to be detrimental to dogs' health, as they act as both definitive and intermediate hosts. Potentially fatal infections may be caused by the larval stage (tethratyridia), which can produce invasive infections in the peritoneum, challenging the ability of clinicians to make diagnosis (16, 17).

Despite the aforementioned advantages of copromicroscopy for the screening of such helminthic infections in domestic animals (e.g., dogs), and the opportunity of non-invasive sampling by red fox environmental stool collection (e.g., for the surveillance of *E. multilocularis*), the sensitivity of copromicroscopic tests for helminthic parasites has rarely been assessed in red foxes (18).

In the perspective of an extended application of copromicroscopy, the present study aimed to first (i) compare the performances of two copromicroscopic tests (i.e., the classical flotation method, for the detection of both cestodes and nematodes; and a filtration/isolation method, followed by a molecular identification of taeniid species performed only on positive samples, that is specific for the detection of cestodes), using the scraping-filtration and counting technique as a reference; and secondly, (ii) to conduct a literature meta-analysis to integrate our findings into a larger perspective.

## 2. Methods

### 2.1. Sampling and laboratory analyses

A total of 150 red fox carcasses were collected across Bolzano province (Italy) by wildlife technicians in the period 2020–2021. Animals were either found dead, within existing passive surveillance programs, or culled during hunting season and population control campaign approved by local wildlife management authorities. Carcasses were transported to the Bolzano Laboratory of Istituto Zooprofilattico delle Venezie and frozen at −80°C for 72 h before examination to inactivate eggs of *Echinococcus* spp., thus preventing infection risk for personnel (19).

At necropsy, a fecal sample was collected from the rectum of each fox using sterile gloves; the small intestine was then tied at both ends, removed and transferred to the Department of Animal Medicine Production and Health, University of Padova. Classical copromicroscopic examination was carried out on separate fecal aliquots by means of two techniques: (i) a classical flotation technique (FT) using a Zinc chloride solution (specific gravity 1.350), followed by observation at optic microscope for identification of eggs at the lowest taxonomic level possible; (ii) a filtration/sieving technique followed by multiplex PCR on positive samples, for identification of taeniid eggs [named CMPCR, as per (20)]. Briefly, to this aim, 2 grams of feces were subjected to flotation; after a centrifugation step, supernatant was passed through 40 and 20 μm mesh sieves and retained eggs were collected. DNA was then extracted, and three couples of primers were initially used to amplify the ND1 gene for *Echinococcus multilocularis* and 12S rRNA for both *E. granulosus* and *Taenia* spp. Positive samples were then amplified using a PCR assay targeting a fragment of the cytochrome oxidase gene and sequenced to obtain species identification as per Citterio et al. (20).

Furthermore, isolation of helminths from the small intestine was performed through scraping, filtration and counting technique (SFCT), which was used as reference test. Briefly, the small intestine was cut into short segments (30 cm) which were longitudinally opened and successively rinsed in tap water to collect all the content in a beaker; the intestinal wall was then scraped, collecting all the washed material in the same beaker. Rinses were then filtered using 1,000 and 212 μm sieves. Non-filtered material was entirely collected and observed under the stereomicroscope (Olympus-SZX12) for the isolation and counting of the helminths. Besides, aliquots corresponding to the 25% of the total volume of washes was examined under the stereomicroscope from the second sieve. Individual parasites were identified morphologically at the lowest taxonomic level possible, using identification keys found in literature (21–30). Briefly, nematodes referred to as members of the superfamily Ascaroidea were identified as *Toxocara* sp. based on the presence of a glandular ventriculus at the end of the esophagus, and the presence of a digitiform-shaped caudal extremity and caudal alae in males, all absent in the genus *Toxascaris*. Moreover, elongated, narrow cervical alae were used as a mark to distinguish the species *T. canis* from *Toxocara cati*, which holds broader and shorter cervical alae. A minimum length 5.6 and 6.1 cm was set as the cut-off to define mature male and female specimens, respectively (21–23). Bursate nematodes were referred to the family Ancylostomatidae. Buccal capsule morphology was observed to distinguish *Uncinaria stenocephala* from *Ancylostoma* spp., considering the presence of two chitinous plates at the ventral border in the former, and one to three developed pairs of ventral teeth, jointly with two dorsal ones, in the latter (22). Moreover, smaller spicules are present in males of *U. stenocephala* (0.64–0.76 μm) compared to *A. caninum* (0.8–0.95 μm) (22). Size, presence of a well-developed bursa and spicula or eggs in uterus were used to confirm sex and sexual maturity (22, 24). Small nematodes of the genus *Pterygodermatites* were distinguished from the aforementioned genera for the presence of two rows of well-developed, peculiar, cuticular spines along the sub-ventral body surface. The presence of eggs in the uteri or unequal spicules was used for the sex determination (25, 26). Presence or absence of rostellum was used to distinguish among Taeniidae and *Mesocestoides* spp., respectively. Morphology and measurements of the hooks was used to identify *Taenia* at species level (27, 28). Lack of a rostellum and presence of four large suckers, together with presence of a central, parauterine organ, were the main characteristics for the identification of *Mesocestoides* sp. (29, 30).

Identification of cestodes at species level was molecularly confirmed by PCR and sequencing. DNA extraction was performed on 7 Taeniidae (*n* = 2 for *Taenia crassiceps, n* = 4 for *Taenia polyacantha* and *n* = 1 for *Hydatigera* [*Taenia*] *taeniaeformis*) and 1 from *Mesocestoides* sp. by using the NucleoSpin^®^ Tissue Kit (Macherey-Nagel, Germany), according to the manufacturer's instructions. The mt-CO1 region was amplified using primers JB3 and JB4.5 as already reported by Bowles et al. (31). PCR amplicons were purified and sequenced from both ends at Macrogen (Macrogen Europe, Amsterdam, The Netherlands). Alignment was performed with Clustal W integrated into MEGA v6.0, and sequences were compared with the non-redundant database available in the GenBank^®^ database using the software BLAST (32).

### 2.2. Data analysis

An agreement table between each of the two copromicroscopic tests (FT and CMPCR) and the scraping one (SFCT) was displayed; the concordance was then calculated for each of the two tests as the number of samples with same result out of the total number of samples examined (% concordance).

The sensitivity (Se) of copromicroscopic tests and 95% confidence interval (95%CI) was assessed for ascarids and Ancylostomatidae (only for FT), and for taeniids (both for FT and CMPCR), using SFCT as the reference standard. Analysis of the sensitivity of the two tests was performed through EpiTools (https://epitools.ausvet.com.au), using the Clopper-Pearson (exact) method for 95% CI definition.

The intensity of infection [i.e., the number of individuals of a particular parasite species in a single infected host, *sensu* Bush et al. (33)], was displayed and descriptively analyzed to assess its influence on the outcome of the copromicroscopic tests.

### 2.3. Literature search and meta-analysis

To unveil the relation between the presence of eggs in the feces and the adults in the intestines, we performed a literature search to complement our findings with data generated by similar studies previously conducted. Prevalence values in other red fox populations were retrieved from literature, focusing on studies conducted exclusively in Europe and using scraping (SCT or SFCT) and copromicroscopic (qualitative flotation or quantitative techniques) approaches on the same animals. For this aim, the database Google Scholar was searched using the terms “copromicroscopic” OR “eggs” AND “red fox” as keywords. Data from single publications investigating different, geographically separated, red fox populations, were considered independently as separated datasets. The level of concordance between the prevalence values obtained with the scraping and the copromicroscopic approaches was assessed through the Bland Altman plot, which graphically describes the agreement between two methods (34). Finally, the pooled prevalence and its 95% CIs were estimated for each parasite taxon, based on the inverse variance method and logit transformation, to account for different sampling sizes among studies (35).

## 3. Results

### 3.1. Copromicroscopic tests performances in the present survey

Overall, SFCT allowed for the detection of parasites from 114 out of 150 foxes (76.0%). Among these, simple infections were present in few cases (*n* = 35; 30.7%). Three species of nematodes (*T. canis, U. stenocephala* and *Pterygodermatites* sp.), and five species of Cestodes (*E. multilocularis, T. crassiceps, T. polyacantha, H. taeniaeformis, Mesocestoides* sp.) were detected and identified, and selected specimens identification was confirmed by molecular analyses (identity >99.0% with *T. crassiceps* [acc.n. OP738082]; 99.7% with *T. polyacantha* [acc.n. OP738083]; 98.4% with *H. taeniaeformis* [acc.n. OP738085]; 99.5% with *Mesocestoides litteratus* [acc.n. OP738084]).

Flotation allowed to detect mixed or simple infections in 79 samples (52.7%). The eggs were referable to *Toxocara* sp., Ancylostomatidae and Taeniidae, as well as to *Trichuris* sp. and *Capillaria* sp., whose adult forms were not detectable by our technique, applied exclusively to the small intestine. No eggs of *Pterygodermatites* sp. or *Mesocestoides* sp. were found with copromicroscopic examination.

As for nematodes, the overall concordance between SFCT and FT was 62.7% for ascarids, and 70.7% for members of the Ancylostomatidae. Most non-concordant values were due to negative results at the FT when adult specimens were found in the intestine with SFCT. In few cases, adult parasites were not detected in the intestine, when ascarids and/or Ancylostomatidae eggs were observed at FT (Table 1). The sensitivity (*Se*) of FT was below 40% for both groups of nematodes. Nevertheless, excluding single-sex infections and infections solely by immature worms, *Se* increased to 46.1% for ascarids and to 42.8% for Ancylostomatidae (Table 1).

**Table 1:** Agreement table between results at SFCT (scraping, filtration and counting technique) and FT (zinc chloride floatation) for ascarids and Ancylostomatidae.

**Agreement table**			**SFCT**			**Concordance %**	**Sensitivity % (95% CI)**	**Corrected sensitivity % (95% CI)**
			**Neg**	**Pos**	**Tot**			
Ascarids	FT	Neg	70	42	112	62.7	36.3 (24.8–49.1)	46.1 (26.5–66.6)
		Pos	14	24	38			
		Tot	84	66	150			
Ancylostomatidae	FT	Neg	95	27	122	70.7	28.9 (15.4–45.9)	42.8 (17.6–71.1)
		Pos	17	11	28			
		Tot	112	38	150			

The number of parasites counted in positive animals at SFCT (intensity) ranged from 1 to 100 for ascarids and from 1 to 32 for hookworms. The intensity of infection did not seem to strongly influence the outcome of the copromicroscopic test, since animals positive at FT were fairly distributed among the different intensities. The highest intensity resulting in a negative copromicroscopic test were 26 ind. for ascarids, and 12 ind. for Ancylostomatidae.

For taeniids, scraping and FT had a low level of concordance (51.3%) that slightly increased for CMPCR (58.7%; Table 2). In the case of cestodes, all positive samples at copromicroscopic tests were confirmed by isolation of cestodes from the intestine, consequently low concordance was only due to false negative results at copromicroscopy (Table 2).

**Table 2:** Agreement table between results at SFCT (scraping, filtration and counting technique) and FT (zinc chloride floatation) and CMPCR (multiplex PCR) for Taeniidae.

**Agreement table**			**SFCT**			**Concordance %**	**Sensitivity % (95%CI)**
			**Pos**	**Neg**	**Tot**		
Taeniidae	FT	Pos	69	73	142	51.3	9.8 (4.3–18.5)
		Neg	0	8	8		
		Tot	69	81	150		
Taeniidae	CMPCR	Pos	69	62	131	58.7	23.5 (14.7–34.2)
		Neg	0	19	19		
		Tot	69	81	150		

Among cestodes, the intensity of infection varied greatly between *Taenia* spp., *Mesocestoides* spp. and *E. multilocularis*, ranging from 1 to 22 for *T. crassiceps* (*n* = 33), from 1 to 82 for *T. polyachanta* (*n* = 31), from 1 to 26 for *Mesocestoides* sp. (*n* = 32), and from 4 to 19,800 for *E. multilocularis* (*n* = 24). A single animal was found infected with a single specimen of *H. taeniaeformis*. Similar to nematodes, the intensity of infection did not seem to strongly influence the outcome of the copromicroscopic test, since animals with highest intensity of adults in the intestine, respectively 22 for *T. crassiceps*, 82 for *T. polyachanta* and 19,800 for *E. multilocularis*, resulted negative at the FT test. As already mentioned, no *Mesocestoides* sp. eggs were detected at the FT.

At CMPCR, eggs referable to taeniids were detected in 19 samples, but only in 13 samples it was possible to identify the species or the genus through Multiplex PCR and sequencing, i.e., *T. crassiceps* (*n* = 7, 1 co-infection), *E. multilocularis* (*n* = 5, 3 co-infections), *T*. *polyacantha* (*n* = 2, 1 co-infection) and *Taenia* sp. (*n* = 2, 1 co-infection). The specific identifications at CMPCR were mostly in agreement with the results of adults' isolation from the intestine (SFCT), as showed in Table 3, although, in some cases of co-infections, one parasite species may remain undetected.

**Table 3:** Number of retrieved adult parasites and specific identification at SFCT (scraping, filtration and counting technique) for the 13 animals positive for Taeniidae molecularly identified at CMPCR (multiplex PCR).

**ID**	**Specific identification at CMPCR**	**Number of adults identified at SFCT**		
		***T. crassiceps***	***T. polyacantha***	***E. multilocularis***
33	*Taenia crassiceps*	22	0	20
37	*Taenia crassiceps*	8	0	0
49	*Taenia crassiceps*	3	11	0
110	*Taenia crassiceps*	8	7	0
171	*Taenia crassiceps*	5	0	0
185	*Taenia crassiceps*	1	0	0
73	*Echinococcus multilocularis + Taenia crassiceps*	5	0	10,572
65	*Echinococcus multilocularis*	0	0	1,456
67	*Echinococcus multilocularis*	0	0	19,800
165	*Echinococcus multilocularis + Taenia polyacantha*	1	0	12
114	*Taenia polyacantha*	1	71	0
197	*Echinococcus multilocularis + Taenia* sp.	0	0	1,116
173	*Taenia* sp.	5	2	0

### 3.2. Analysis of prevalence data from literature

**Figure 1:**
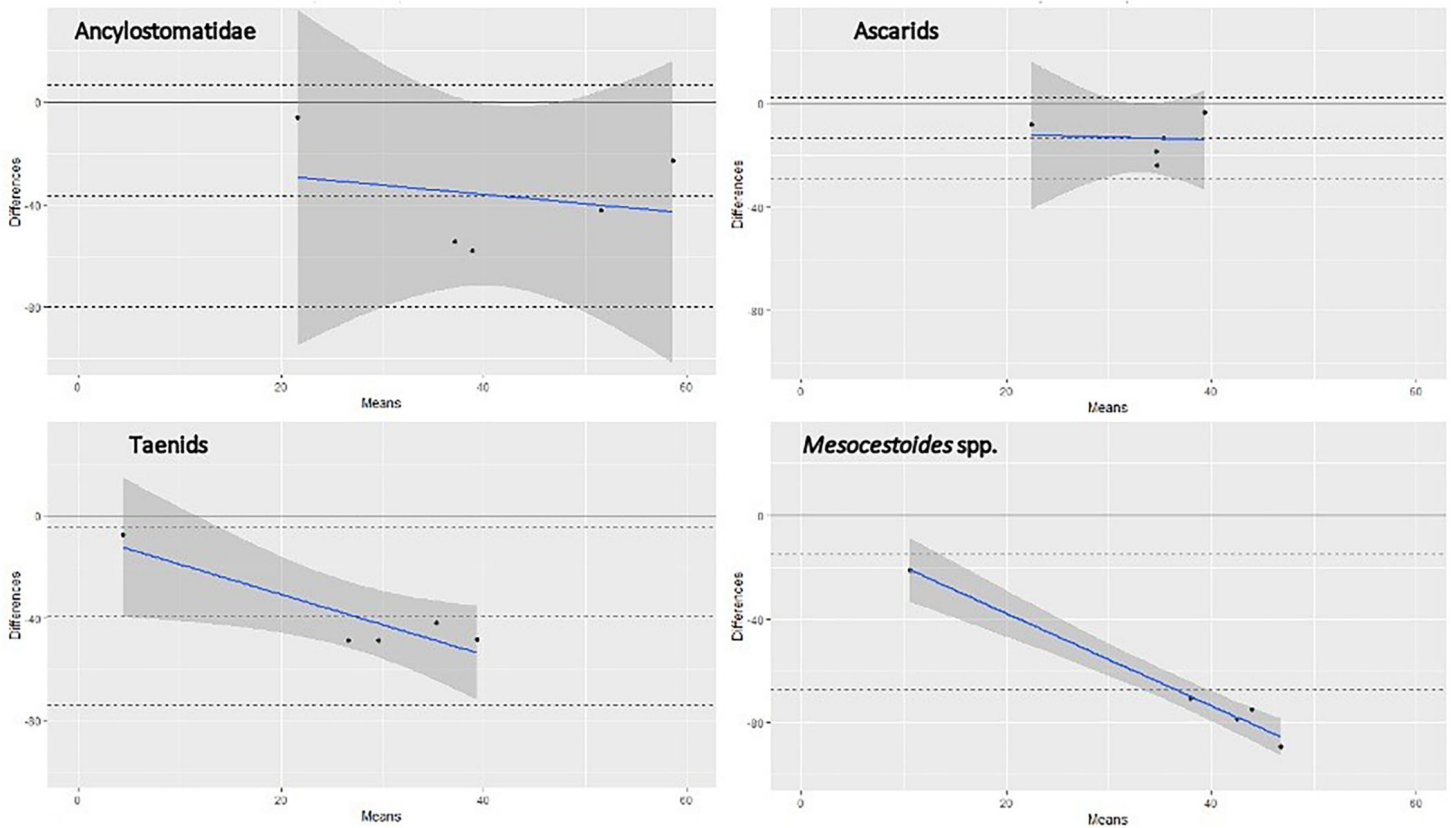
Bland-Altman graph representing the agreement between SFCT (reference test) and copromicroscopy, for four different groups of helminths, based on literature data and those from the present study. Note: each dot represents one pair of prevalence values (scraping and copromicroscopic) reported in Table 4, identified by the mean of the two values in the x-axis and by the difference between the two values in the y-axis. The continuous gray line at the 0 value in the y-axis corresponds to the prevalence value at scraping (reference test), whereas the central dashed line is the mean difference of the copromicroscopic value. The continuous blue line shows the trend of the differences between values, at increasing mean prevalence values. Refer to the main text for references used in this graph.

Based on the literature search, we found five studies investigating helminthic infection in red fox populations using, at the same time, scraping and copromicroscopic techniques (18, 36–39). However, three studies (36, 38, 39) were excluded from the analysis because the prevalence values at necropsy and at copromicroscopy were calculated on different subsets of animals, and the prevalence values in the group of animals analyzed by both methods were not reported. In one study, the investigated red fox population was split in three different datasets, due to their geographical separation. Therefore, a total of five datasets, including ours, were used in the meta-analysis. The prevalence values reported in each study for each considered group of parasites are listed in Table 4, along with the estimated pooled prevalence, and their concordance is displayed in Figure 1, using a Bland-Altman graph.

**Table 4:** Prevalence values estimated using scraping and copromicroscopic methods in four datasets retrieved from literature and in the present study.

**Study area**	**Sample size (*n*)**	**Scraping** **method**	**Copromicroscopic (Copro)** **method**	**Ancylostomatidae**		**Ascarids**		**Taeniidae**		***Mesocestoides*** **sp**.		**References**
				**Scraping Prev (%)**	**Copro Prev (%)**	**Scraping Prev (%)**	**Copro Prev (%)**	**Scraping Prev (%)**	**Copro Prev (%)**	**Scraping Prev (%)**	**Copro Prev (%)**	
NW Italy	180	SCT	Flotation Zinc sulfate (s.g., 1.35)	70.0	47.2	26.7	18.3	8.3	0.6	81.7	3.3	Magi et al. (18)
SE Poland	140	SCT	Flotation magnesium sulfate (s.g., 1.22)	64.3	10	42.1	28.6	56.4	14.3	91.4	2.1	Karamon et al. (36)
N Poland	92	SCT	Flotation magnesium sulfate (s.g., 1.22)	72.8	30.4	46.7	22.8	51.1	2.2	81.5	6.5	Karamon et al. (36)
NE Poland	112	SCT	Flotation magnesium sulfate (s.g., 1.22)	67.9	9.8	41.1	37.5	63.4	15.2	73.2	2.7	Karamon et al. (36)
NE Italy	150	SFCT	Flotation zinc sulfate (s.g., 1.35)	24.7	18.7	44.0	25.3	54.0	5.3	21.3	0.0	This study
Pooled prevalence % (95%CI)				59.8 (55.8–63.7)	27.9 (24.3–31.8)	39.3 (35.6–43.1)	26.3 (23.1–29.8)	49.5 (45.3–53.7)	10.6 (8.0–13.8)	68.3 (63.9–72.4)	3.4 (2.2–5.3)	

The prevalence value was constantly lower when estimated by copromicroscopy, rather than scraping, with mean difference ranging from about 15% for ascarids, to around 40% in the case of Ancylostomatidae and taeniids (Figure 1). *Mesocestoides* was the most underestimated genus at copromicroscopy, since the mean difference in prevalence values reaches about 70%. Moreover, the difference in the estimates between the two approaches increased with increasing prevalence values, although this was evident for cestodes, and less clear for nematodes. These differences were similarly highlighted by the calculation of the pooled prevalence, which was always higher at necroscopy (Table 4).

## 4. Discussion

Our study aimed at assessing the performances of two copromicroscopic techniques in the estimation of presence and prevalence of gastrointestinal helminths of the red fox, including the ones of veterinary and public health interest. Although extensive work has been conducted on the different diagnostic approaches for the detection of *E. multilocularis* in red foxes, few studies addressed the performances of copromicroscopic analyses for other helminthic species, especially nematodes.

In our study, the prevalence values obtained for all taxa with the copromicroscopic approach were invariably lower than the values estimated by scraping, though variation in the extent of such deviation is found among taxa, with cestodes being the most underestimated. The sensitivity of the two copromicroscopic methods used in this study was indeed low for all helminths, ranging from 9.8 to 46.1%, with minimum values for taeniids. Similarly, prevalence of helminthic infections in red fox populations appeared to be always underestimated in the studies comparing copromicroscopic and scraping approaches, with reductions of about 50% for nematodes and from 5-fold to 20-fold decreases for cestodes (Table 4). The extent of such underestimation increased along with the increase in prevalence, at least for cestodes (Table 4, Figure 1).

In our case, the widest difference in prevalence estimates was found for cestodes, and for *Mesocestoides* spp. in particular, whose eggs may go completely undetected, or occur with 20-fold lower prevalence than that obtained through the scraping of intestines (36). This may be explained by the peculiarity of the egg structure in *Mesocestoides* spp., being this fragile and rapidly inactivated after the release from the proglottids (40). Consequently, visualization of whole proglottids in fresh fecal samples probably represents a better way to evaluate the presence of the species with respect to egg detection through flotation (41). As for taeniids, inconstant shedding of proglottids is the reason for frequent false negative results at copromicroscopic diagnosis of these parasites. This was confirmed by the low sensitivity of simple flotation in fecal samples of foxes found in our study for taeniids (9.8%), which was surprisingly similar to that estimated (9.0%) by Magi and colleagues (18). The concentration of taeniid eggs from fecal material in the CMPCR procedure is achieved through flotation followed by filtering and eggs collection which increases the sensitivity of the coprological diagnosis for taeniid infection, as also demonstrated by this study, with a 23.5% sensitivity. Specific identification of taeniid eggs, through the following steps of Multiplex PCR and sequencing, was concordant in almost all cases with species isolated from the intestines, confirming again the accuracy of the assay.

The low sensitivity of copromicroscopy for nematode infections in the red fox was common also in in literature. Regardless of the copromicroscopic technique used, several authors reported prevalence estimation for Ancylostomatidae to be up to 10-fold lower with copromicroscopy (36, 37). Such a low performance using red fox fecal samples may be partially due to storage conditions. Indeed, safety protocols for the inactivation of *E. multilocularis* eggs with the deployment of temperature require deep freezing at −80°C for at least 48 h (19). The thin egg walls of Ancylostomatidae are known to be particularly sensitive by freezing, resulting in distortion and unrecognizable morphology, or even rupture (42). Similarly, we can expect that the thin-walled eggs of *Pterygodermatites* sp. undergo similar important modifications, explaining the lack of detection by copromicroscopy. Alternative effective safety protocols for the inactivation of *Echinococcus* spp. eggs include the use of chemicals, with glutaraldehyde being the only effective (19, 43), but toxicity of this compound makes this alternative quite questionable for extensive application. Thus, deep freezing is almost unavoidable and, realistically, the consequent bias has to be simply taken into account. The thick-walled eggs of *Toxocara canis* and Taeniidae, on the other side, seem to be unaffected by deep freezing if not in their color or aspect of the internal morula, which should not prevent identification (42). Nevertheless, the sensitivity of copromicroscopic methods for *Toxocara* spp. eggs was reported to be modest when performed on dogs and cats feces as well, underestimating the occurrence of this helminth in the intestine of these species of up to 50% with a Zinc-sulfate flotation (44), and also with McMaster technique (45), when no freezing protocols had been applied.

In our study, we observed that copromicroscopy overlooked parasitic eggs in animals with high numbers of mature specimens found at SFCT, suggesting that the correlation between adult parasites intensity and fecal egg count still need further investigations. Given the overall limited performances of traditional and partly modified copromicroscopic techniques (e.g., FT and CMPCR), the adoption of different approaches for the analysis of fecal material, such as the use of molecular test alone for all collected samples, should be at least considered. The use of real-time PCR has led indeed to a further important increase in the sensitivity of the copromicroscopic test for the detection of *E. multilocularis* (46–50) allowing for the detection of low-intensity infections.

However, the application on large-scale surveys of molecular methods would carry evident economic and logistic costs and required the availability of equipped laboratories (51). Such an approach would be appropriate for helminthic species causing severe zoonoses, such as *Echinococcus* spp., but it may not be sustainable for other parasitic species. Therefore, leaving behind the traditional copromicroscopic approach, information about the diversity of the helminth community may get partially lost.

In conclusions, this study highlighted the ability of two copromicroscopic approaches to detect the presence in red fox feces of the main helminthic taxa with zoonotic and/or veterinary importance. Eggs of all taxa isolated at SFCT, with exclusion of *Mesocestoides* sp. and *Pterygodermatites* sp., were indeed detected through these techniques. Nevertheless, their sensitivity was demonstrated to be critically low for all taxa, and mostly for taeniids, resulting in a constant underestimation of the prevalence in the investigated populations, as also supported by the literature. Anyway, the drawbacks of the copromicroscopic techniques should not exclude *a priori* their use in monitoring and surveillance activities, but their low sensitivity has to be considered when conducting large-scale surveys aimed at defining the prevalence of helminthic infections in a given population. The expected underestimation of the real prevalence should be taken into consideration while interpreting the results. At the same time, surveys aimed at the detection of the parasites must consider the estimated sensitivity of the tests used, to allow for a correct definition of the sample size.

## Data availability statement

The datasets presented in this study can be found in online repositories. The names of the repository/repositories and accession number(s) can be found in the article.

## Author contributions

EM performed morphological and molecular analyses, contributed to the conception of the study, and wrote the first draft of the manuscript. FO, RCe, PD, and CVC performed necroscopic and laboratory analyses and contributed to the conception of the study. LC, AMas, and FM contributed to the conception of the study. AMau contributed to the conception of the study and performed part of the statistical analyses. RCa contributed to the conception of the study, performed statistical analyses, and wrote sections of the manuscript. All authors contributed to manuscript revision, read, and approved the submitted version.
