# Corrigendum: Comparing copromicroscopy to intestinal scraping to monitor red fox intestinal helminths with zoonotic and veterinary importance

**DOI:** 10.3389/fvets.2023.1190058

**Published:** 2023-05-11

**Authors:** Erica Marchiori, Federica Obber, Roberto Celva, Federica Marcer, Patrizia Danesi, Anna Maurizio, Lucia Cenni, Alessandro Massolo, Carlo Vittorio Citterio, Rudi Cassini

**Affiliations:** ^1^Department of Animal Medicine, Production and Health, University of Padova, Legnaro, PD, Italy; ^2^Istituto Zooprofilattico Sperimentale delle Venezie, Legnaro, PD, Italy; ^3^Ethology Unit, Department of Biology, University of Pisa, Pisa, Italy; ^4^Applied Ecology Research Unit, Research and Innovation Centre, Fondazione Edmund Mach, Trento, Italy; ^5^Conservation Genomics Research Unit, Research and Innovation Centre, Fondazione Edmund Mach, Trento, Italy; ^6^Department of Ecosystem and Public Health, Faculty of Veterinary Medicine, University of Calgary, Calgary, AB, Canada; ^7^UMR CNRS 6249 Chrono-Environnement, Université Bourgogne Franche-Comté, Besançon, France

**Keywords:** copromicroscopy, gastro-intestinal parasites, *Echinococcus multilocularis*, helminths, red fox

In the published article, there was an error regarding the affiliation for “Lucia Cenni.”

As well as having affiliation “^3^ Ethology Unit, Department of Biology, University of Pisa, Pisa, Italy,” she should also have had affiliations “^4^ Applied Ecology Research Unit, Research and Innovation Centre, Fondazione Edmund Mach, Trento, Italy” and “^5^ Conservation Genomics Research Unit, Research and Innovation Centre, Fondazione Edmund Mach, Trento, Italy.”

The authors apologize for this error and state that this does not change the scientific conclusions of the article in any way. The original article has been updated.

